# Loss of the *PTCH1* tumor suppressor defines a new subset of plexiform fibromyxoma

**DOI:** 10.1186/s12967-019-1995-z

**Published:** 2019-07-30

**Authors:** Sudeep Banerjee, Christopher L. Corless, Markku M. Miettinen, Sangkyu Noh, Rowan Ustoy, Jessica L. Davis, Chih-Min Tang, Mayra Yebra, Adam M. Burgoyne, Jason K. Sicklick

**Affiliations:** 1 Division of Surgical Oncology, Department of Surgery, Moores Cancer Center, University of California, San Diego, UC San Diego Health Sciences, 3855 Health Sciences Drive, Room 2313, Mail Code 0987, La Jolla, CA 92093-0987 USA; 2 Department of Surgery, David Geffen School of Medicine at UCLA, Los Angeles, CA USA; 3 Department of Pathology and Knight Cancer Institute, Oregon Health & Science University, Portland, OR USA; 4 Laboratory of Pathology, National Cancer Institute, Bethesda, MD USA; 5 Division of Hematology Oncology, Department of Medicine, Moores Cancer Center, University of California, San Diego, La Jolla, CA USA

**Keywords:** Submucosal tumor, Gastric mass, Patched 1, GLI1, Hedgehog pathway, SMO inhibitor, Sonidegib, Gastrointestinal stromal tumor, Next generation sequencing

## Abstract

**Background:**

Plexiform fibromyxoma (PF) is a rare gastric tumor often confused with gastrointestinal stromal tumor. These so-called “benign” tumors often present with upper GI bleeding and gastric outlet obstruction. It was recently demonstrated that approximately one-third of PF have activation of the *GLI1* oncogene, a transcription factor in the hedgehog (Hh) pathway, via a MALAT1-GLI1 fusion protein or GLI1 up-regulation. Despite this discovery, the biology of most PFs remains unknown.

**Methods:**

Next generation sequencing (NGS) was performed on formalin-fixed paraffin-embedded (FFPE) samples of PF specimens collected from three institutions (UCSD, NCI and OHSU). Fresh frozen tissue from one tumor was utilized for in vitro assays, including quantitative RT-PCR and cell viability assays following drug treatment.

**Results:**

Eight patients with PF were identified and 5 patients’ tumors were analyzed by NGS. An index case had a mono-allelic *PTCH1* deletion of exons 15–24 and a second case, identified in a validation cohort, also had a *PTCH1* gene loss associated with a suspected long-range chromosome 9 deletion. Building on the role of Hh signaling in PF, PTCH1, a tumor suppressor protein, functions upstream of GLI1. Loss of PTCH1 induces GLI1 activation and downstream gene transcription. Utilizing fresh tissue from the index PF case, RT-qPCR analysis demonstrated expression of Hh pathway components, *SMO* and *GLI1*, as well as GLI1 transcriptional targets, *CCND1* and *HHIP*. In turn, short-term in vitro treatment with a Hh pathway inhibitor, sonidegib, resulted in dose-dependent cell killing.

**Conclusions:**

For the first time, we report a novel association between *PTCH1* inactivation and the development of plexiform fibromyxoma. Hh pathway inhibition with SMO antagonists may represent a target to study for treating a subset of plexiform fibromyxomas.

**Electronic supplementary material:**

The online version of this article (10.1186/s12967-019-1995-z) contains supplementary material, which is available to authorized users.

## Background

Plexiform fibromyxoma (PF) is a rare submucosal gastric tumor with unknown incidence that can be confused with myxoid gastrointestinal stromal tumors (GIST) [1]. While slow growth and lack of metastases suggest an indolent natural history, these so-called “benign” tumors often present with upper gastrointestinal bleeding and gastric outlet obstruction. Although the cell of origin remains unknown, PF generally occurs in the gastric muscularis propria and frequently invades into the mucosa and submucosa [1–4]. Histologically, PF is characterized by a multinodular plexiform growth pattern, diffuse myxoid stroma and prominent thin arborizing capillaries. Immunohistochemically, PF is characterized by expression of α-SMA (alpha smooth muscle actin) while lacking expression for CD117 (KIT, c-KIT), DOG-1 (discovered on GIST-1), CD34, S-100, desmin (or focal), and cytokeratins. On this basis, PF can be differentiated from GIST, as well as leiomyoma, leiomyosarcoma, schwannoma, gastric carcinoids, and desmoids [1, 3].

To date, there has been only one report investigating the molecular biology of PF. The authors demonstrated that 5 of 16 tumors had activation of the *GLI1* oncogene, a transcription factor in the hedgehog (Hh) pathway [5]. Two tumors were found to have *GLI1* amplification, while three had *MALAT1*-*GLI1* oncogenic fusions. Both types of *GLI1* genomic alterations resulted in overexpression of GLI1 protein and activation of Hh signaling. This highly conserved pathway has been implicated in the biology of several types of cancers, including gastroblastoma [6], basal cell carcinoma [7], medulloblastoma [8], rhabdomyosarcoma [9], hepatocellular carcinoma [10], and GIST [11], as well as tumors with plexiform and fibromyxoid histologies [12]. The pathway is activated when the hedgehog ligands, namely sonic hedgehog (SHH), indian hedgehog (IHH) and desert hedgehog (DHH) ligands, bind to their receptor, Patched 1 (PTCH1), a multi-pass transmembrane protein. In an unbound state, PTCH1 negatively regulates smoothened (SMO), a seven-transmembrane domain proto-oncoprotein. Following ligand binding, the PTCH1 tumor suppressor protein releases SMO inhibition, which then leads to activation of the GLI family of transcription factors that includes the GLI1 proto-oncoprotein. In turn, GLI1 regulates expression of many genes involved in cell cycle progression, including Cyclin D1 (*CCND1*), as well as members of the Hh pathway itself, including PTCH1, GLI1, and hedgehog-interacting protein (HHIP) [13]. Thus, oncogenic mutation of *SMO* or *GLI1*, as well as inactivating mutations of *PTCH1* can activate the pathway and downstream transcription [13]. However, only SMO and PTCH1-altered tumors can be targeted with the three FDA-approved SMO inhibitors, namely sonidegib (Novartis/Sun), vismodegib (Genetech-Roche), and glasdegib (Pfizer).

The *PTCH1* tumor suppressor gene is located on the long arm of chromosome 9 (9q22.32). Over 500 different *PTCH1* mutations have been implicated in Gorlin syndrome (i.e., basal cell carcinoma and rhabdomyosarcoma), sporadic basal cell carcinoma, holoprosencephaly, keratocystic odontogenic tumors, and ocular developmental anomalies [14]. Herein, we present the first report of recurrent *PTCH1* loss in plexiform fibromyxoma based on next generation sequencing. This newly identified genetic alteration is the first tumor suppressor associated with PF tumorigenesis. In turn, we examined the role of *PTCH1* inactivation on Hh signaling and investigated a rational approach to pharmacologically treating *PTCH1*-, but not *GLI1*-mutated PF, with SMO inhibitors.

## Methods

### Human subjects

Written informed consent was obtained for all study participants, including publication of clinical data. Patient tissue collection, acquisition of clinical data, and conducting experimental procedures on biological samples was approved or exempt by each institutional IRB [UC San Diego Human Research Protections Program Institutional Review Board (IRB) Protocol #090401, NCI Office of Human Subjects Research (OHSR) IRB exemption was granted for work with anonymized annotated human samples, and OHSU IRB exemption was granted for contribution of one patient to the study cohort]. All experiments were conducted in accordance with appropriate regulatory guidelines for use of human tissue. Excess explant tissue was collected for study. An index case from UCSD served as the initial observation for this study. Additional patients were retrospectively identified from the NCI and OHSU. These were analyzed in a validation cohort.

### Pathologic diagnosis

Pathologic diagnosis was performed at each institution. In general, pathologic diagnosis of PF requires histopathology showing spindle shaped cells, diffuse or focally plexiform architecture, prominent thin capillaries and a bland myxoid background. Additionally, immunohistochemical staining is characteristically positive for α-SMA while negative for CD117, CD34, DOG-1, and S-100.

### Primary tumor dissociation and single cell suspension

Excess fresh tumor tissue was dissociated into single cell suspensions using the gentleMACS Dissociator (Miltenyi Biotec, San Diego, CA) as previously described [15]. Solid tissues were cut into 5-mm size pieces and were transferred to a gentleMACS C-Tube containing RPMI 1640 media and MACS human tumor dissociation enzyme solution (Miltenyi Biotec) according to manufacturer’s instructions for tough tumor tissue (h_Tumor_01). The sample was then passed through a 70 μm filter, and tumor cells were collected following centrifugation. The single cells were cultured in RPMI 1640 supplemented with 20% fetal bovine serum (FBS), 1% penicillin/streptomycin (Mediatech, Manassas, VA) and 2 mM glutamine (Mediatech).

### Next generation sequencing

Sequencing was performed on formalin-fixed paraffin-embedded (FFPE) sections (6 tumors from National Cancer Institute (NCI), 1 tumor from Oregon Health & Science University (OHSU) and 1 tumor from University of California, San Diego (UCSD). FoundationOne-Heme Next Generation Sequencing (NGS) was performed on Tumor 1 as previously described [16]. Briefly, DNA was extracted from FFPE sections with a minimum of 20% tumor tissue. DNA was submitted for comprehensive genomic profiling with hybridization-captured, adaptor ligation-based libraries. The FoundationOne-Heme test interrogates a cancer-related library of 400 gene-associated genes, 30 introns and 250 RNA transcripts tailored for hematologic malignancy and sarcoma [17]. Tumors 2–5 were analyzed by the GeneTrails Comprehensive Solid Tumor Panel (Knight Diagnostic Laboratories, OHSU), which is a DNA sequencing panel that screens for alterations in 124 known oncogenes and tumor suppressor genes. DNA extracted from FFPE tumor tissue must provide a minimum of 50 ng DNA content, which is required to achieve a minimum depth of 250 reads per amplicon. Samples that had less than this minimum required DNA content did not undergo NGS analysis. In addition, Tumors 2–5 were subjected to RNA sequencing using the GeneTrails Solid Tumor Fusion Gene Panel (Knight Diagnostic Laboratories, OHSU), which is a partner-agnostic assay for gene fusions across 22 cancer-related target genes. RNA extracted from FFPE tumor tissue required a minimum of 30 ng RNA content for adequate analysis. Samples with less than the required amount were not analyzed by RNASeq.

### RNA preparation and expression quantification

Total RNA for in vitro experiments was prepared from single cell suspension using the RNeasy Mini Kit (Qiagen, Hilden, Germany). RNA quality was assessed with the Nanodrop 2000 (Thermo Scientific, Waltham, MA). Reverse transcription quantitative real-time polymerase chain reaction (RT-qPCR) was performed on a CFX96 real-time system (Bio-Rad Laboratories, Hercules, CA) using SsoFast EvaGreen Supermix (Bio-Rad Laboratories). Each sample was tested in triplicate. Forward and reverse primer sequences are as previously described [10, 18]. The threshold cycle (Ct) of target genes were normalized to *ACTB* according to delta-Ct method [18]. PCR transcript expression and appropriate size were validated by gel electrophoresis using a 2.0% agarose gel in 1× Tris–acetate-EDTA buffer.

### Cell viability assay

Single cell suspensions of tumor cells were seeded at 2500 cells per well on a 96-well plate (Corning, Lowell, MA). The cells were grown for 48 h and subsequently treated with Sonidegib (LDE225, Novartis, Basel, Switzerland) with a titration of 50-, 25-, 12.5- and 6.25-μM for 72 h based upon prior reports in mantle cell lymphoma, rhabdomyosarcoma, and chronic myeloid leukemia [19–21]. The CellTiter-Glo Luminescent Cell Viability Assay (Promega, Madison, WI) was performed and luminescence measured on the Tecan Infinite 200 microplate reader (Tecan, Männedorf, Switzerland).

### Statistical analysis

Statistical analyses were performed using Prism GraphPad 7 (GraphPad Software, La Jolla, CA). Results are expressed as mean ± standard deviation. Comparison were made using student’s *t*-test and statistical significance was accepted at the 5% level. Inhibitor concentration-50 (IC_50_) was calculated using GraphPad 7.

## Results

### Patient demography

Overall, eight cases of pathology confirmed plexiform fibromyxoma were examined from three institutions. An index case (Tumor 1) served as the initial observation and then seven additional patients were retrospectively identified and analyzed in a validation cohort (Table 1). Overall, the median age at diagnosis was 45.5 years old (range 16–65 years). There were five females (62.5%) and three males in the cohort. Tumor locations were distributed throughout the stomach, except one tumor that was described as originating from the duodenum. The median tumor size was 3.5 cm (range 2.8–11 cm).

**Table 1:** Demographic and clinical characteristics of the cohort

Patient	Age	Sex	Tumor location	Size (cm)	Institution	Altered chromosome	Genes in deletion
1^a^	65	M	Stomach, antrum	5.0	UCSD	9	*PTCH1* deletion exons 15–24
2	61	F	Duodenum	11.0	NCI	9	*PTCH1* *FANCC*
3	16	M	Stomach	Unknown	NCI	11 and 12	*CHEK1* (Chr11) *CDKN1B* (Chr12) *DDX11* (Chr12) *ERBB3* (Chr12)
4	43	F	Stomach, distal	3.0	NCI	N/A	None detected^b^
5	48	M	Stomach	2.8	OHSU	N/A	None detected^b^
6	49	F	Stomach	3.5	NCI	N/A	Failed QC
7	15	F	Stomach, prepyloric	6.0	NCI	N/A	Failed QC
8	32	F	Stomach, antrum	3.0	NCI	N/A	Failed QC

*N/A* not applicable, *NCI* National Cancer Institute, *OHSU* Oregon Health & Science University, *QC* quality control, *UCSD* University of California, San Diego

^a^Patient 1 represents an index case observation

^b^No gene deletions detected within the GeneTrails panel of 124 known oncogenes and tumor suppressor genes. Does not rule out the possibility of gene losses in those not included in the panel

### Next generation sequencing (NGS)

DNA and RNA extraction were performed on FFPE samples for all tumors. DNA extraction met minimum quality requirements in 4 out of 8 samples (50%) and RNA extraction met minimum quality requirements in 5 out of 8 samples (62.5%). NGS was performed by either the FoundationOne-Heme Panel (Tumor 1, Foundation Medicine) or GeneTrails Comprehensive Solid Tumor and Fusion Gene Panels (Tumors 2–5, Knight Diagnostic Laboratories, OHSU). Overall, 3 out of 5 samples had alterations in cancer-related genes and no gene fusion products were detected (Table 1).

#### Index case

##### Tumor 1

**Fig. 1:**
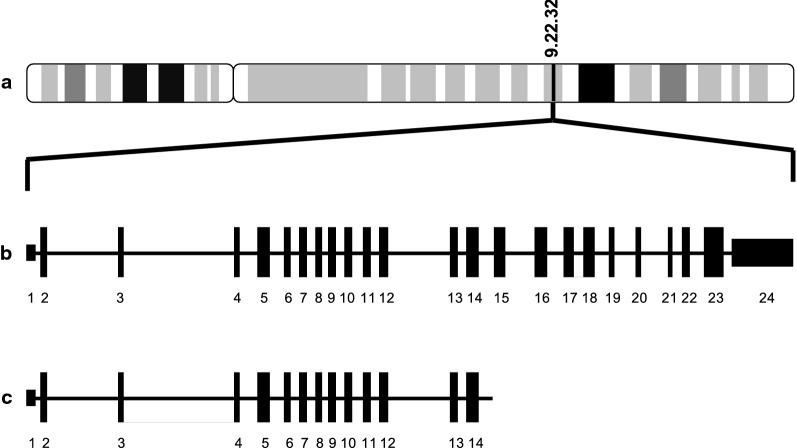
*PTCH1* gene schematic. **a** *PTCH1* origin on q arm of chromosome 9, **b** full length transcript with 24 exons and **c** mutant *PTCH1* with deletion of exons 15–24 (Adapted from https://www.genecards.org and https://www.atlasgeneticsoncology.org, respectively)

NGS identified a partial *PTCH1* deletion of exons 15–24 on chromosome 9q (Fig. 1). This mutation corresponds to a partial loss of the 4th extracellular loop (ECL) and complete loss of the 5th and 6th ECLs, as well as loss of transmembrane domains 8 thru 12, the 4th and 5th intracellular loops (ICLs), and the C-terminus cytoplasmic domain. This predicts a loss of tumor suppressor function due to altered inhibition of the SMO proto-oncoprotein. In addition, the tumor also had genomic alterations in *NOTCH1* L2457V (designated as a benign genomic alteration in ClinVar) and *NOD1* L475fs*35. Nucleotide-binding oligomerization domain-containing protein 1 (NOD1) involved in the recognition of bacterial molecules and stimulation of an immune reaction [22]. Thus, we suspect that these are passenger mutations.

#### Validation cohort

##### Tumor 2

Tumor 2 had bi-allelic chromosome 9q deletions of *PTCH1* and *FANCC* (Fanconi anemia complementation group C). *FANCC* encodes a protein involved in homologous recombination repair of damaged DNA and delaying apoptosis. Whether loss of FANCC played a role in the development of this tumor is unclear. Examination of the involved chromosomal loci (Chr9: 95,099,054–95,517,057 from GRCh38/hg38 in UCSC Genome Browser [23]) shows that *FANCC* and *PTCH1* are the only coding genes located within this position. Loss of both *FANCC* and *PTCH1* suggests a long-range deletion of chromosome 9 that likely encompassed at least partial deletions of both genes. However, it is certainly possible that the deletion encompassed a larger region that was not detected by the GeneTrails panel.

##### Tumor 3

A third tumor was found to have bi-allelic chromosome 12 deletions of *CDKN1B*, *DDX11* and *ERBB3*. CDKN1B encodes p27^Kip1^, which regulates cell cycle progression by inhibiting cyclin E-CDK2 and cyclin D-CDK4. The *DDX11* gene encodes a DNA/RNA helicase that has myriad functions in maintenance of genomic stability, chromosomal organization in mitosis and translation initiation. *ERBB3* encodes HER3 (human epidermal growth factor receptor 3), which has not been found to be independently oncogenic but forms active heterodimers with ErbB2. The loci of genes affected [*CDKN1B* (12p13.1), *DDX11* (12p11.21), and *ERBB3* (12q13.2)] are distributed throughout chromosome 12 suggesting that individual alterations, not long-range deletions, occurred. Furthermore, proximate and intervening genes such as *CCND2* (12p13.32), *KRAS* (12p12.1) and *MDM2* (12q15) were found to be intact, also suggesting that the chromosome 12 deletions were partial (or full) gene deletions of individual loci rather than long-range deletions. Tumor 3 also had bi-allelic loss of *CHEK1* on chromosome 11. Checkpoint kinase 1 (Chk1), the protein product of *CHEK1,* is involved in surveillance of genomic integrity and is necessary for initiation of the DNA damage checkpoint. We suspect that *CHEK1* (11q24.2) was altered by a partial or full-length deletion rather than a long-range deletion because an neighboring gene, *SDHD* (11q23), was intact. However, a long-range deletion that encompassed other genes than *CHEK1*, but were not assessed by the GeneTrails panel, cannot be excluded.

##### Tumors 4–5

Two tumors did not have any cancer-associated genetic alterations detected on NGS by the GeneTrails panel (124 known oncogenes and tumor suppressor genes), although this does not rule out that other genes may be involved in tumorigenesis.

##### Tumors 6–8

Three tumors did not meet minimum requirements for DNA quality for analysis by NGS.

### Hh pathway is expressed in PTCH1-inactivated PF

**Fig. 2:**
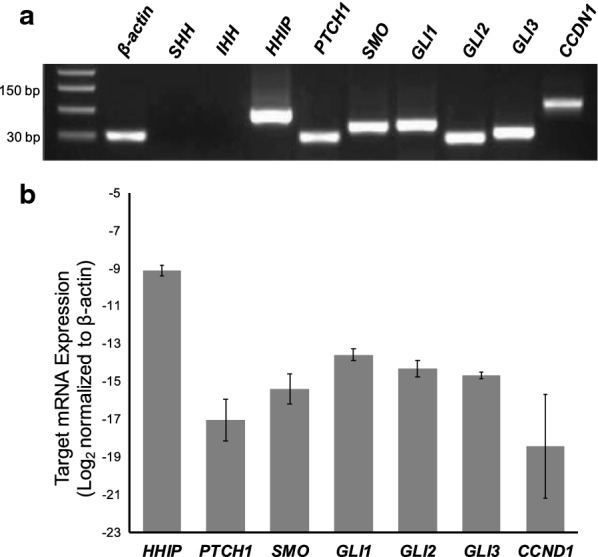
Hedgehog pathway is expressed in *PTCH1*-inactivated plexiform fibromyxoma. **a** Validation of transcript PCR products on agarose gel electrophoresis (cropped as shown; full length gel is presented in Additional file 1: Figure S1) and **b** relative quantification of expressed hedgehog pathway components using the delta-Ct method. Experiments performed in triplicate (N = 3)

Tumors 1 and 2 both had genetic alterations causing loss of the *PTCH1* gene. To assess whether *PTCH1* deletion was associated with activation of Hh signaling, we performed quantitative RT-PCR analysis of Hh pathway components and Hh target genes. Fresh tumor tissue was available from Tumor 1 (index case) and was utilized for in vitro assays. This analysis demonstrated mRNA expression of Hh pathway components (*PTCH1*, *SMO*, *GLI1, GLI2* and *GLI3*) as well as downstream GLI1 transcriptional targets (*CCND1* and *HHIP*) (Fig. 2). It is noteworthy that *PTCH1* and *GLI1* are both Hh pathway components and are also GLI1 transcriptional targets. In the tumor, the Hh pathway ligands, *SHH* and *IHH*, had undetectable expression consistent with ligand-independent Hh pathway activation following *PTCH1* inactivation (Additional file 1: Figure S1).

### PTCH1-inactivated PF is sensitive to targeted Hh inhibition

**Fig. 3:**
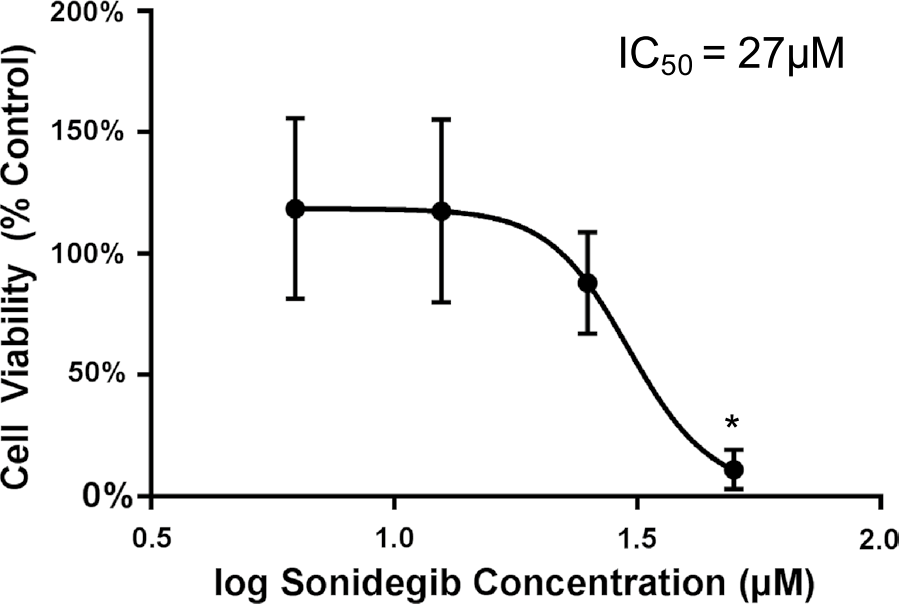
Dose-dependent cell death in *PTCH1*-inactivated PF following SMO inhibitor treatment. Four-point dose titration of sonidegib treatment. Absolute experimental and control data shown in Additional file 1: Figure S2. Experiments performed in triplicate (N = 3). *P < 0.001

We next performed cell killing assays with a SMO proto-oncoprotein inhibitor to assess the cellular dependency on Hh signaling. Viable tissue from Tumor 1 was dissociated into single cell suspension and plated in cell culture. Treatment of the primary tumor cells with the Hh pathway inhibitor, sonidegib (LDE225, Novartis), resulted in dose-dependent cell killing with an IC_50_ of 27 μM (Fig. 3 and Additional file 1: Figure S2). Drug dose range was selected for IC_50_ finding based on literature cited values [19–21]. Thus, targeting *PTCH1*-mutant PF cells with a SMO inhibitor resulted in tumor cell death.

## Discussion

In a recent study, *GLI1* upregulation through a *MALAT1*-*GLI1* fusion or copy number amplification was detected in a subset of PF [5]. Spans et al. demonstrated the critical nature of Hh signaling in this disease. Here, we identified two cases of plexiform fibromyxoma (PF) with genomic loss of *PTCH1*, a tumor suppressor gene in the hedgehog pathway. Quantitative PCR of one of these tumors supported ligand-independent activation of the Hh pathway. In addition, targeting smoothened in the context of *PTCH1* inactivation resulted in cell killing of primary PF cells. This approach represents the first in vitro use of a targeted therapeutic strategy for this rare tumor.

Our new findings lend further support for a primary role of aberrant Hh signaling in PF pathophysiology. In contrast to the previously reported *GLI1* alteration, we observed loss of *PTCH1*, indicating that events occurring upstream in the Hh pathway can also drive PF growth [5]. Although it is possible that non-canonical activation of the Hh pathway accounts for the expression of Hh pathway components, we believe this is unlikely since non-canonical Hh activation usually results in low SMO levels whereas *Smo* mRNA was found to be high in Tumor 1 [24, 25]. We also show that targeted SMO inhibition in vitro, which lies downstream of PTCH1, but upstream of GLI1, leads to dose-dependent cell killing of PF cells. This supports the role of PTCH1/SMO-axis oncogenesis since non-canonical Hh activation is SMO-independent and usually resistant to SMO inhibition. In contrast to *PTCH1*-mutated PF, *GLI1* upregulated PFs are predicted to be resistant to SMO inhibition. Thus, our new findings provide evidence for a possible therapeutic role of Hh inhibition in the treatment of *PTCH1*-inactivated PF. Additional testing using in vivo models will be necessary to determine whether SMO inhibition is a viable strategy for treating patients with *PTCH1*-inactivated PF.

Hedgehog signaling has been associated with several tumor types. In fact, germline *PTCH1* mutations underlie nevoid basal-cell carcinoma syndrome, also known as Gorlin Syndrome. This is an autosomal dominant condition associated with distinctive facial abnormalities, as well as, basal cell carcinomas and mesenchymal tumors. Interestingly, histopathologic features of tumors associated with Gorlin Syndrome bear striking similarities to plexiform fibromyxoma. For example, Gorlin-associated odontogenic myxofibrous tumors have a bland myxoid appearance in a plexiform pattern [26]. Similarly, a subset of pediatric gastric pericytomas have been associated with an *ACTB*-*GLI1* gene fusion [27]. Additionally, six cases of malignant epithelioid neoplasms with frequent S100-positivity were all found to have *GLI1* fusions involving *ACTB*, *MALAT1* or *PTCH1* [28]. These tumors may represent a histologically novel class of pericytoma or soft tissue sarcoma distinct from plexiform fibromyxoma. Collectively, these observations underscore a strong genotypic-to-histopathologic/morphologic relationship in *PTCH1* associated diseases.

In addition to syndromic diseases, *PTCH1* mutations have been implicated in several cancers including sporadic basal cell carcinoma (BCC) [29], squamous cell carcinoma [30], medulloblastoma [8], and embryonal rhabdomyosarcoma [31]. There are over 500 *PTCH1* mutations described in human disease [14]. The mono-allelic exon 15–24 deletion of *PTCH1* in Tumor 1 would be predicted to truncate the C-terminal region. Interestingly, elegant mutagenesis experiments in *Drosophila* have shown that the C-terminus of *ptch1* is necessary for inhibition of the Hh pathway and that deletion of the last 156 residues produces a ligand-independent dominant negative phenotype [32, 33]. Consistent with these data, *PTCH1* haploinsufficiency has been reported in variety of cancer types, including basal cell carcinoma, medulloblastoma, and rhabdomyosarcoma [34]. Furthermore, “one-hit” *PTCH1* inactivation has been described in up to one-third of patients with nevoid basal cell carcinoma syndrome (NBCCS) who have mono-allelic *PTCH1* mutations [35]. Taken together, this suggests that single allele inactivation of *PTCH1* can have a dominant negative effect and may account for Hh activation, as well as tumorigenesis, in a subset of PF.

Based on the prior observation of *GLI1* overexpression in PF and the present identification of *PTCH1* inactivation, it appears that there are two distinct subsets of PF identified to date. It is interesting to note that the previously reported *MALAT1*-*GLI1* fusion protein implicates chromosomal loci 11q13 (*MALAT1*) and 12q13 (*GLI1*). Tumor 3 from our cohort showed loss of *ERBB3* (12q13; close to *GLI1*) and *CHEK1* (11q24). Unfortunately, the RNA from this case was too degraded to screen for a possible *GLI1* fusion.

There are several limitations to our study. First, primary cells were expanded in culture for several days before performing cell killing assays. Therefore, it is possible that these cells do not fully recapitulate in vivo biology. Second, the PF cells were not immortalized and we were limited in the amount of in vitro studies that could be performed before viable cells were exhausted. Thus, additional mechanistic studies including genetic *PTCH1* rescue experiments, *SMO* knockdown experiments, and assessment of Hh target gene expression following SMO inhibitor treatment were not feasible. Development of an immortalized PF cell line would prove an extremely useful tool in further characterization of PF pathophysiology.

## Conclusions

We report a novel association between *PTCH1* inactivation and the development of plexiform fibromyxoma. Our findings demonstrate that tumor suppressor gene alterations (i.e., *PTCH1*), rather than oncogenic mutations (i.e., *GLI1*), within the Hh pathway are also associated with plexiform fibromyxoma. In turn, targeted Hh pathway inhibition with SMO antagonists may represent a target to study for treating a subset of plexiform fibromyxomas. Further studies are warranted to investigate the clinical efficacy of these agents in appropriately selected patients, as well as to determine the underlying biology of tumors lacking *PTCH1* and *GLI1* alterations.

## Additional files

**Additional file 1: Figure S1.** Validation of transcript PCR products on agarose gel electrophoresis (full length gel).

**Additional file 2: Figure S2.** Absolute experimental and control data for cell viability assay.

## Data Availability

All permissible protected heath information is included in Table 1 on the clinical data of the cohort. In vitro data is available on request. No additional datasets or materials were generated or utilized in this study.

## Abbreviations

PF: plexiform fibromyxoma
PTCH1: Patched 1
SMO: smoothened
Hh: hedgehog
α-SMA: alpha smooth muscle actin
DOG-1: discovered on GIST-1
SHH: sonic hedgehog
IHH: indian hedgehog
DHH: desert hedgehog
CCND1: cyclin D1
HHIP: hedgehog-interacting protein
NGS: next generation sequencing
RT-qPCR: reverse transcription quantitative polymerase chain reaction
IC50: inhibitory concentration 50%
GIST: gastrointestinal stromal tumor
IRB: Institutional Review Board
FFPE: formalin-fixed paraffin-embedded
ECL: extracellular loop
ICL: intracellular loop
BCC: basal cell carcinoma
NBCCS: nevoid basal cell carcinoma syndrome
