# Supplementary figures and images for: Loss of the PTCH1 tumor suppressor defines a new subset of plexiform fibromyxoma
Source: J Transl Med. 2019 Jul 30;17:246. doi: 10.1186/s12967-019-1995-z (PMC6668176; doi:10.1186/s12967-019-1995-z)

***β-actin***

***SHH***

***IHH***

***HHIP***

***PTCH1***

***SMO***

***GLI1***

***GLI2***

***GLI3***

***CCDN1***

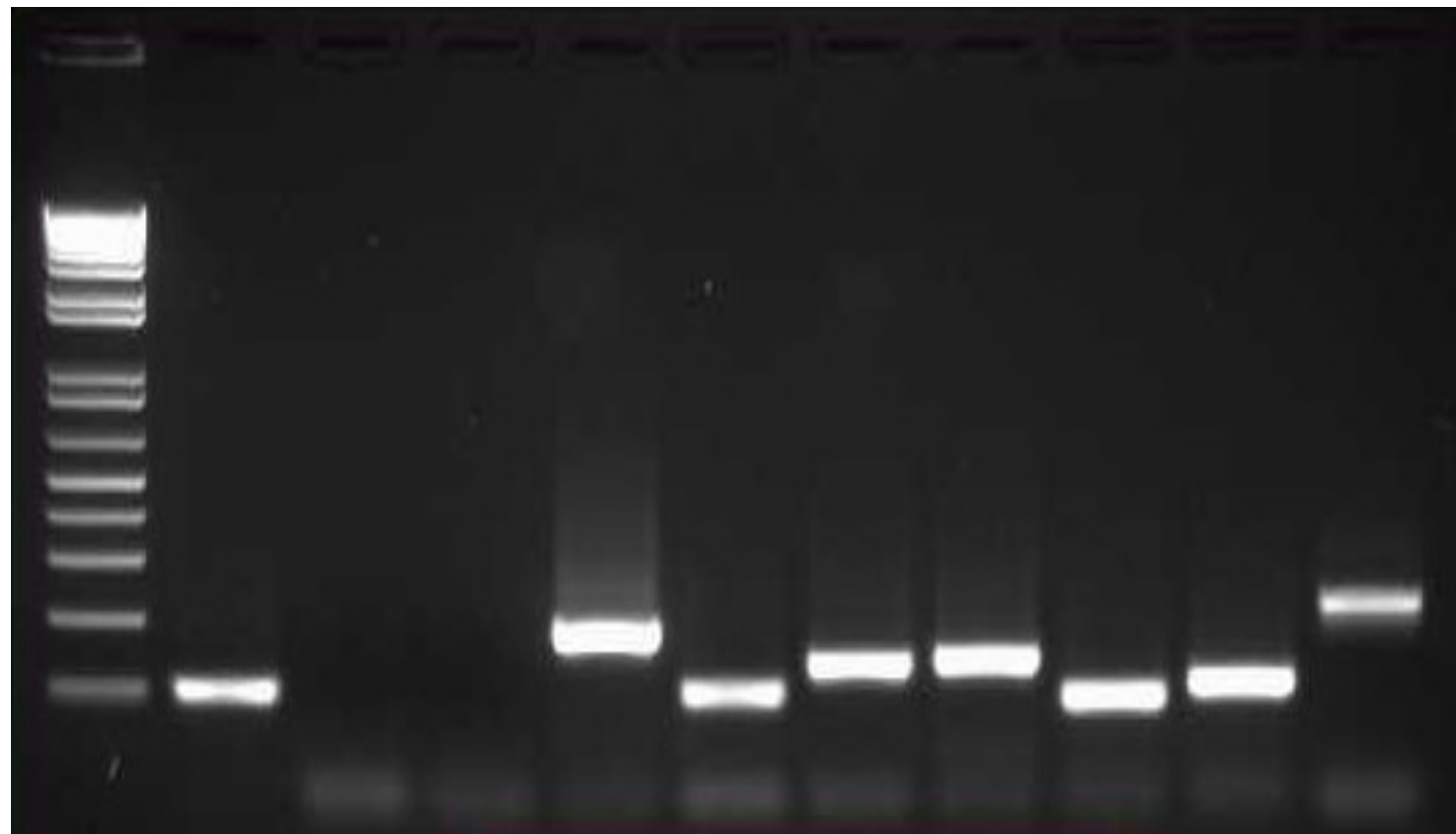

Supplement: Supplementary file 1 — Additional file 1: Figure S1. Validation of transcript PCR products on agarose gel electrophoresis (full length gel). [file 12967_2019_1995_MOESM1_ESM.pdf]

**Luminescence Units  
(CellTiterGlo)**

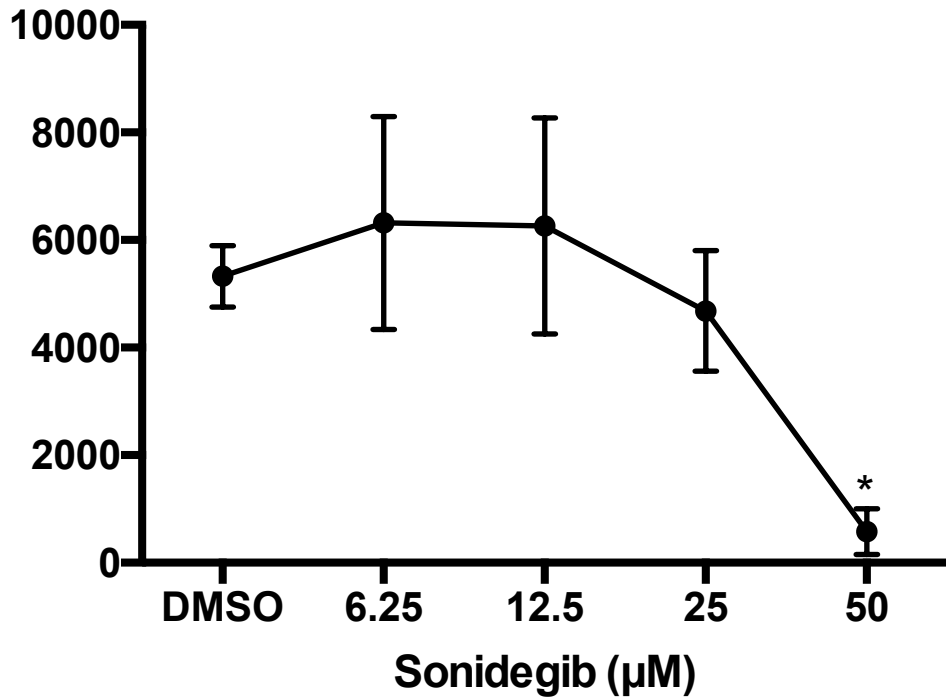

Supplement: Supplementary file 2 — Additional file 2: Figure S2. Absolute experimental and control data for cell viability assay. [file 12967_2019_1995_MOESM2_ESM.pdf]
